# Twisting graphene into correlation and topology

**DOI:** 10.1093/nsr/nwag363

**Published:** 2026-06-12

**Authors:** Shuo-Ying Yang, Cheng Shen

**Affiliations:** State Key Laboratory of Quantum Functional Materials, Department of Physics, and Guangdong Basic Research Center of Excellence for Quantum Science, Southern University of Science and Technology, Shenzhen 518055, China; Quantum Science Center of Guangdong-Hong Kong-Macao Greater Bay Area, Shenzhen 518045, China; School of Physics, University of Electronic Science and Technology of China, Chengdu 610054, China

**Keywords:** twisted graphene, moiré superlattice, superconductivity, topology, electronic correlation

## Abstract

Twisted graphene moiré superlattices have emerged as a highly tunable platform in which electronic correlation and band topology intertwine in unprecedented ways. Near the magic angle, moiré interference dramatically suppresses kinetic energy, producing flat electronic bands with nontrivial quantum geometry and multiple internal degrees of freedom. These features enable interaction-driven phases that have no direct analogue in conventional solids. In this Review, we survey recent progress on magic-angle twisted bilayer graphene and related twisted multilayer graphene systems, focusing on how many-body and topological effects reconstruct the flat-band spectrum and organize the observed phase diagram. We discuss correlated insulating states, flavor-selective cascades, intervalley coherent orders, and the coexistence of localized moments with itinerant carriers. We further examine the emergence of topological phases—including orbital Chern insulators, fractional Chern insulators, and topological electronic crystals—that arise from the interplay between interactions and band topology. Superconductivity in twisted graphene is reviewed as a strongly coupled, unconventional phenomenon, shaped by both electronic correlation and quantum geometry. The discussion is then extended to twisted multilayer graphene architectures, highlighting the intertwined electronic correlation and band topology, and their influence on superconductivity. We conclude by outlining key open problems in superconductivity and opportunities for engineering novel correlated and topological states in twisted graphene moiré systems.

## Teaser

This review highlights how stacking layers of graphene at small angle creates unique flat electronic bands, enabling electrons to interact in ways that unlock completely new and unexpected quantum states of matter.

## INTRODUCTION

Electron–electron interactions play an important role in shaping the behavior of quantum materials, often driving these systems into emergent phases that lie beyond the prediction of single-particle theories. When interactions are sufficiently strong, the collective dynamics of electrons can stabilize exotic states of matter, including unconventional superconducting states with nontrivial pairing mechanisms, Mott insulating states arising from interaction-induced localization, and topological phases characterized by fractionalized excitations [1–3]. These phenomena manifest as a direct consequence of the interplay between kinetic energy, Coulomb repulsion, and underlying lattice or band structure, leading to highly nontrivial many-body correlations. The profound theoretical and experimental implications posed by these systems—ranging from the breakdown of conventional Fermi-liquid descriptions to the emergence of long-range entanglement and novel quasiparticles—underscore why correlation physics remains a central and continuously evolving theme in condensed matter research [3,4].

**Figure 1.**
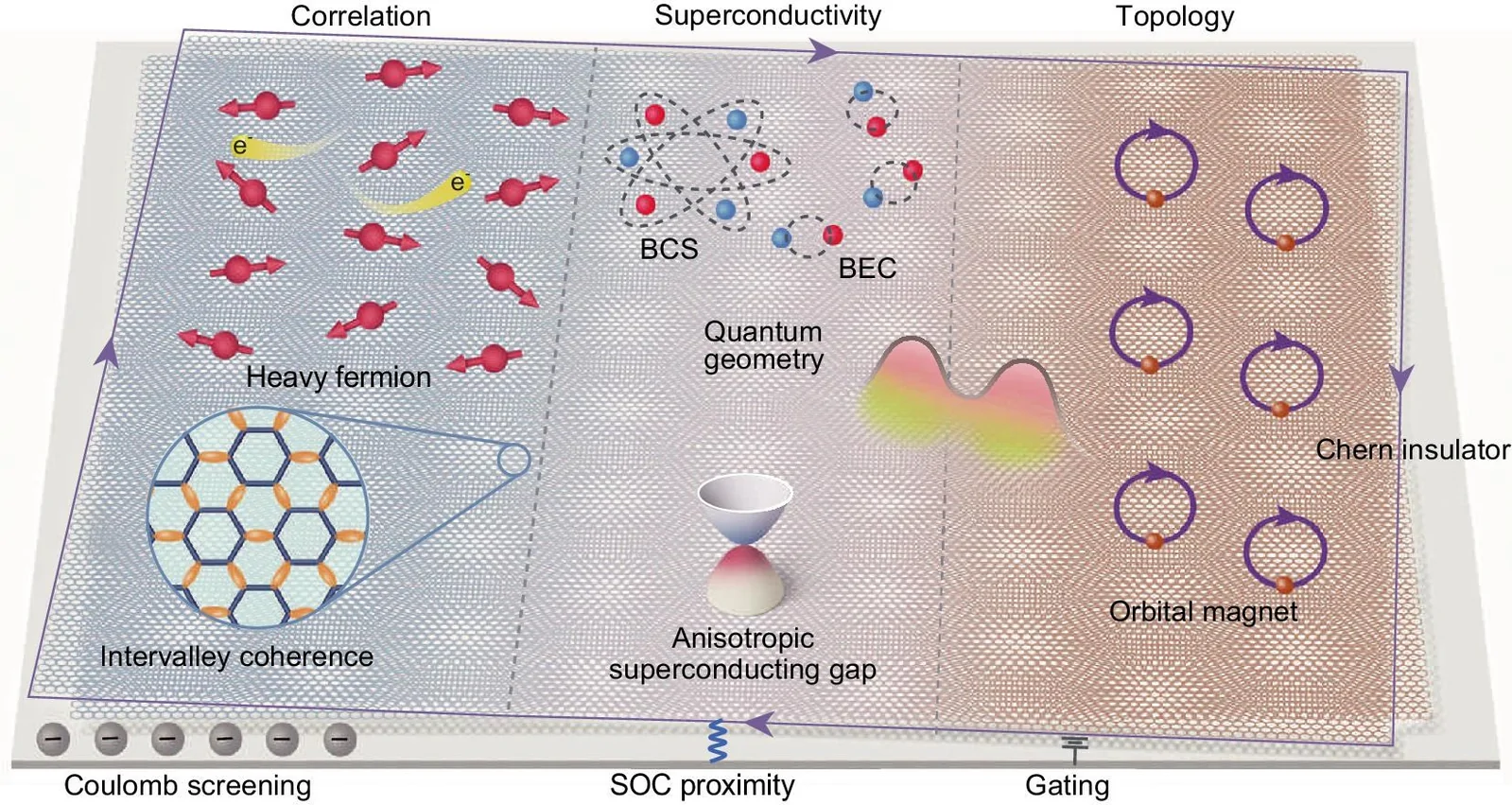
Schematic overview of quantum states that are associated with electronic correlation and topology in twisted graphene moiré superlattice. Superconductivity is depicted as being simultaneously intertwined with both correlation and topology.

The advent of moiré engineering in van der Waals heterostructures has opened a new frontier in the study of strongly correlated and topological quantum materials. By introducing a slight twist between two atomically thin layers, long-wavelength moiré superlattices emerge, giving rise to highly tunable electronic structures. These moiré patterns can drastically reshape the band dispersion, leading to flat bands that amplify electron–electron interactions and enable a wide range of emergent quantum phenomena. Recent progress has revealed a diverse landscape of novel quantum phenomena in moiré materials (Fig. 1). These include unconventional superconductivity, quantum phase transition, Chern insulators, orbital ferromagnets, fractional Chern insulators (FCI) and so on—all of which have markedly surpassed conventional theoretical paradigms [5–12]. Moiré systems offer a uniquely tunable platform for exploring correlated electronic phenomena, providing easier-to-access control over band filling, displacement field, and twist angle compared to traditional quantum materials.

Among the broader class of moiré systems, twisted graphene moiré superlattice stands out as the seminal platform that gave rise to the field of “twistronics.” Hosting a multicomponent subset of isospin flavors and nontrivial quantum geometry, twisted graphene systems exhibit rich and distinctive moiré flat-band physics that is intimately associated with the interplay of topology, electronic correlation, symmetry breaking and superconductivity.

This review is organized around three central concepts—twist, correlation and topology—and their interplay in influencing the emergent quantum phases, particularly superconductivity. From an experimental perspective, we are trying to cover a broad scope of moiré physics with a focus on magic-angle twisted bilayer graphene (MATBG). This review begins with a discussion on how the twist angle acts as a band-engineering knob to generate moiré flat bands with strongly suppressed kinetic energy in MATBG. We then examine the many-body reconstruction of these flat bands, highlighting flavor symmetry breaking, intervalley coherent order, and other interaction-driven phenomena revealed by advanced real-space, momentum-space, and thermodynamic probes. Next, we review the emergence of topological phases, including orbital Chern insulators, FCIs, and topological electronic crystals, arising from the interplay between electronic correlations and band topology. We further discuss the non-BardeenCooperSchrieffer (BCS) superconductivity in MATBG, emphasizing its strong-coupling nature, gap structure, and the role of quantum geometry in the superfluid response. The scope is then extended to twisted multilayer graphene systems, which exhibit enhanced tunability and new correlated behaviors. Finally, we outline key open questions and future directions concerning the microscopic origin of superconductivity and the exploration of novel quantum phases in twisted graphene systems.

## TWIST AS A BAND-ENGINEERING KNOB

**Figure 2.**
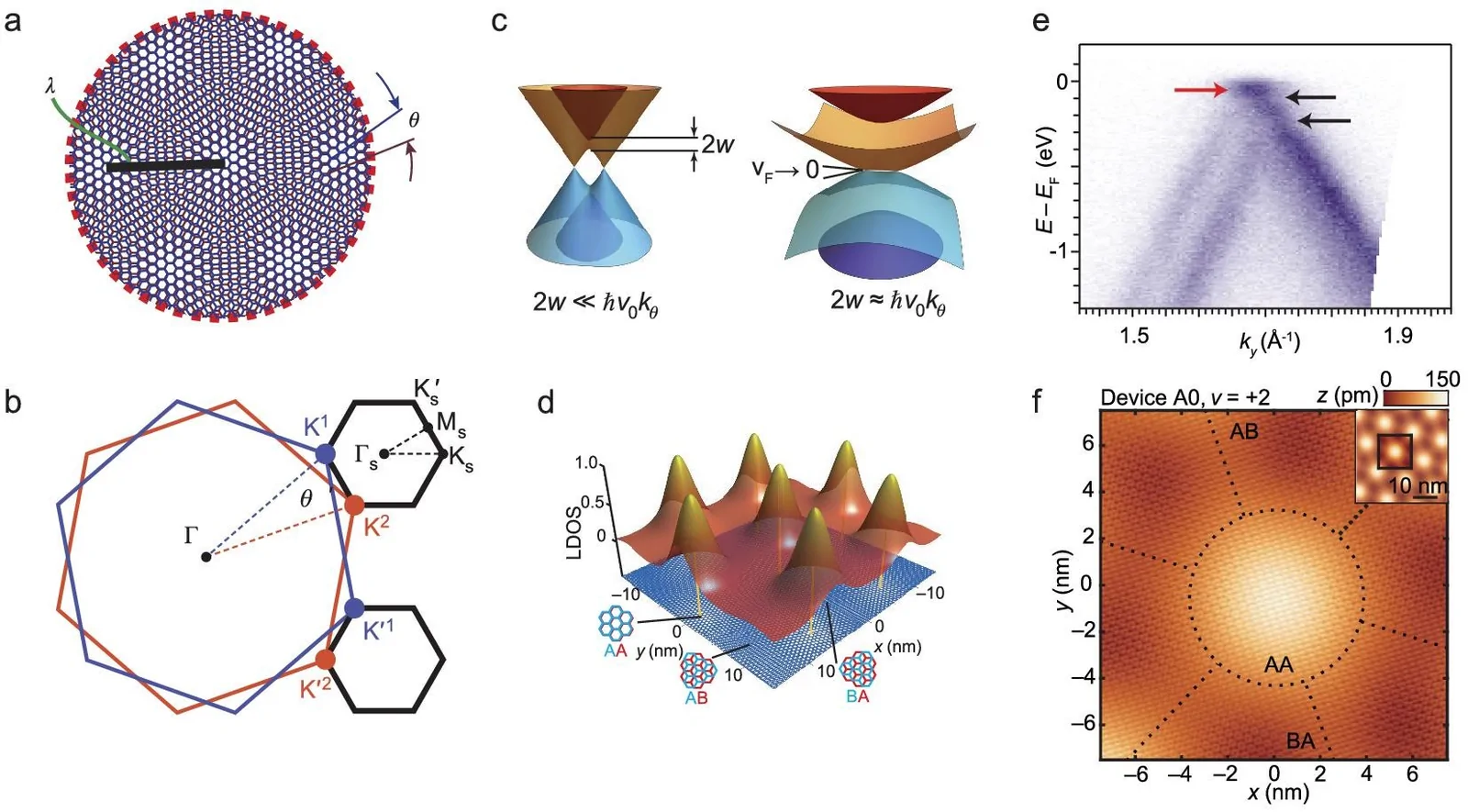
Emergence of flat band in MATBG [5]. (a) Moiré pattern formed in MATBG, where *θ* is the twist angle between two graphene layers. (b) Mini-Brillouin zone arising from the relative rotation of the two Dirac cones, defined by the mismatch between the K points of the top and bottom layers. (c) Illustration of the effect of interlayer hybridization. When the hybridization energy *2w* is smaller than the kinetic energy scale $\hbar {{v}_0}{{k}_\theta }$, the two layers remain effectively decoupled. As *2w* approaches $\hbar {{v}_0}{{k}_\theta }$, significant interlayer hybridization occurs, leading to band reconstruction and the emergence of the flat band. (d) Local density of states (LDOS) under magic angle condition. Electron density is strongly localized in the AA stacking region, while significantly suppressed in the AB and BA stacking areas. (e) Nano-ARPES measured energy band structure of MATBG, in which the flat band and multiple hybridization gaps are marked by red and black arrows respectively [17]. (f) Atomic topography of MATBG measured at *ν = 2* in MATBG by STM [25]. The dashed circle surrounds the AA region, and the radial dotted lines indicate the bridge regions that separate AB and BA regions.

Twisting two periodic lattices against each other at a small angle creates large-scale periodic interference-like patterns, the so-called moiré pattern, shown in Fig. 2a. The moiré structures exhibit a wavelength *λ* that is inversely proportional to the twist angle *θ*, typically given by


(1) \begin{eqnarray*}
\lambda \approx \frac{a}{{2\sin \left( {\frac{\theta }{2}} \right)}},
\end{eqnarray*}

where *a ≈ 0.246* nm is the lattice constant of graphene.

To the zeroth order, the low-energy band structure of twisted bilayer graphene can be considered as two sets of monolayer-graphene Dirac cones rotated about the ${\mathrm{\Gamma }}$ point in the Brillouin zone by the twist angle *θ*. The difference between the two K (or K′) wavevectors gives rise to the mini-Brillouin zone, as shown in Fig. 2b [13]. The resulting band structure behavior of twisted bilayer graphene is governed by the interplay between two energy scales: the strength of interlayer coupling *w* and the band separation energy. One can introduce a dimensionless parameter $\alpha = \frac{w}{{\hbar {{v}_0}| {{{k}_\theta }} |}}$ to characterize the degree of band reconstruction, where *w* is the interlayer potential, ${{v}_0}$ is the Fermi velocity of graphene and ${{k}_\theta }$ is the wavevector of the moiré pattern. Two distinct regimes of *α* are associated with different behaviors. At large twist angle (or small *α*), the layers remain weakly coupled and preserve linear dispersion of each individual Dirac cone (Fig. 2c left). As the twist angle decreases, the Dirac cones near either the K or K’ valley mix through interlayer hybridization, whereas interactions between distant Dirac cones are suppressed exponentially (Fig. 2c right). As a result, Fermi velocity gets renormalized. The renormalized Fermi velocity ${{v}_f}( \alpha )$ can be expressed as:


(2) \begin{eqnarray*}
{{v}_f} = \left| {\frac{{\partial E( k)}}{{\partial k}}} \right| = v_f^0\frac{{1 - 3{{\alpha }^2}}}{{1 + 6{{\alpha }^2}}},
\end{eqnarray*}

where $v_f^0$ is the monolayer graphene Fermi velocity. As can be seen, for small values of *α*, ${{v}_f}$ decreases slightly, indicating a minor modification to the linearly dispersive bands. However, as *α* keeps increasing, ${{v}_f}$ decreases rapidly until it reaches zero at $\alpha = \frac{1}{{\sqrt 3 }}$. The special angle corresponding to ${{v}_f} = 0$ is the so-called magic angle where flat band physics emerge. At the first magic angle ${{\theta }_1} = \frac{{\sqrt 3 w}}{{\hbar {{v}_F}{{G}_k}}} \approx 1.1$°, the Fermi velocity at the mini-Brillouin zone corners drops to zero, resulting in electronic states becoming highly localized in momentum space [13,14]. These electronic states, associated with the flat bands, also exhibit real-space localization at AA stacking regions within the moiré superlattice [13,15,16], as illustrated in Fig. 2d.

Direct experimental evidence for these flat bands has been provided by nano angle-resolved photoemission spectroscopy (ARPES) measurements and scanning tunneling microscopy (STM). Nano ARPES experiments reveal a strong concentration of spectral weight near the moiré Brillouin zone corners, as well as multiple hybridization gaps that signal the formation of moiré minibands [17]. The bandwidth of the flat bands is found to be on the order of tens of millielectronvolts, in good agreement with theoretical expectations (indicated by the red arrows in Fig. 2e) [17–19]. The STM topographies of MATBG, shown in Fig. 2f, reveal a moiré superlattice in which the bright (dark) regions correspond to the AA (AB/BA) stacking regions that are associated with high (low) local density of states (LDOS). By tuning the carrier concentration via the gate modulation, STM enables observation of the dynamic evolution of moiré Bloch bands with respect to charge filling.

**Figure 3.**
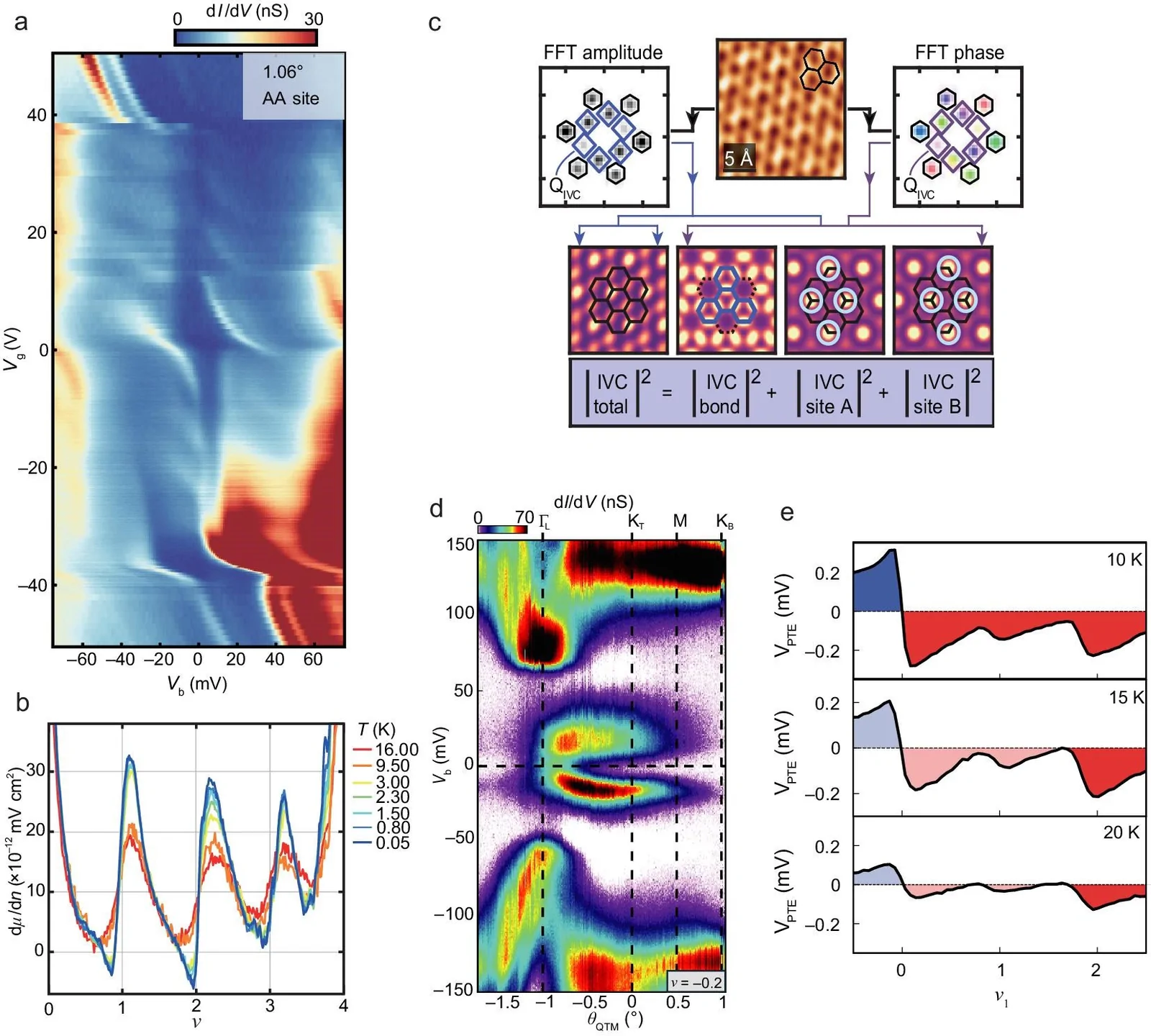
Characterization of electronic correlation in the flat band of MATBG: (a) STM measured differential conductance d*I*/d*V* of the cascade of transitions in MATBG [26]. Reorganization of the low-energy excitations of MATBG happens near each integer fillings of the moiré flat bands. (b) Inverse compressibility of the cascade of transition in MATBG, measured using scanning single-electron transistor [27]. The characteristic sawtooth signal reflects chemical potential resets near integer fillings. (c) STM topographic image acquired at filling factor *ν* = −2 in MATBG reveals atomic-scale signatures of the IVC ground state [25]. A high-resolution region of the image is analyzed to extract the local FFT amplitude and phase, which are then decomposed into three IVC order parameters. (d) Momentum resolved spectroscopy of MATBG measured by QTM. Key momenta are marked on the top axis [45]. At ${{K}_T}$, the measurement discloses two extremely flat bands, separated by a large energy gap. The gap appears at almost all momentum except near the *Γ* point, where the bands are gapless. (e) Temperature-dependent thermoelectric response for a MATBG device at 10 K (top), 15 K (middle) and 20 K (bottom) [46]. As lattice temperature decreases, the response evolves from conventional sign-preserving towards sign-preserving thermoelectricity at integer fillings, indicating electron-hole asymmetry in electron-doped correlated states.

At charge filling where the flat bands are fully occupied or empty, STM resolves the van Hove singularities associated with nearly flat conduction and valence bands as two sharp peaks whose energy separation is consistent with a noninteracting model [20–24]. Once the chemical potential is tuned into the flat bands, STM spectra exhibit pronounced energy broadening of the flat band features, reflecting the strong Coulomb interaction (Fig. 3a) [21–24].

## CORRELATION: MANY-BODY RECONSTRUCTION OF FLAT BANDS

The strongly reduced bandwidth endows MATBG with pronounced electronic correlation that profoundly affects its electronic behavior. In this section, we highlight the key features of correlated electronic behavior and discuss the underlying many-body physics associated with symmetry breaking, phase transition, and electron localization.

### Correlated insulators and flavor symmetry breaking

Owing to the spin and valley degrees of freedom in graphene, complete filling of each degenerate valence and conduction flat band in MATBG requires a total of four electrons or holes per moiré unit cell. The band-filling factor *ν*, defined as the number of electrons and holes per moiré unit cell, ranges from -4 when all flat bands are empty, to 0 corresponding to charge neutrality with the valence flat bands filled, and up to 4 when all flat bands are filled. According to single-particle Bloch band theory, a partially filled valence or conduction flat band (-4 < *ν* < 0 or 0 < *ν* < 4) is expected to be metallic. However, experimental results show pronounced gapped states at filling factor *ν* = ±2 and *ν* = 3, providing solid evidence of electronic correlation in MATBG [5,7,8]. Generally, if the kinetic energy of electrons is less than the on-site Coulomb interaction in a spin-degenerate Bloch band, the nearest electron hopping is forbidden, giving rise to an antiferromagnetic Mott insulating state at half filling. Like a Mott insulator, the suppressed Fermi velocity of the flat band in MATBG reduces kinetic energy to a scale comparable with the Coulomb interaction which is inversely proportional to the moiré wavelength. Consequently, correlated insulating states appear at these integer fillings.

Electronic correlation in MATBG drives spontaneous spin and valley flavor symmetry breaking. Adding carriers to the system, the spin and valley flavors are populated not equally but through a sequence, forming a cascade of phase transitions. Within the flat bands, spectroscopic measurements of STM (Fig. 3a) reveal sharp transitions at every integer filling, indicating a cascade of electronic phase transitions that are attributed to Hubbard sub-bands with lifted spin and valley degeneracies [26]. Thermodynamic measurements that can manifest electron correlation, energy gaps, or phase transitions as anomalies or suppressions in electronic compressibility provide more details on the cascade of phase transitions [26–29]. In scanning single-electron transistor (SET) thermodynamic measurements as shown in Fig. 3b, a striking sequence of sawtooth-like features in compressibility appears near every integer filling [27]. The asymmetric sudden jump and a subsequent gradual decrease in compressibility correspond to a Dirac-like electronic character as near the charge neutrality point (CNP). When the carrier density changes approaching every integer filling, a single flavor takes carriers from other flavors, leaving other flavors unoccupied and forming a ‘reset’ state back to charge neutrality. The reset state is in line with the Landau-level asymmetry reported in magneto-transport measurements [8], where Landau fan from nonzero integer fillings emanate in only one direction away from charge neutrality. These Dirac revival behaviors are also accompanied with negative compressibility—manifested as the pinning of the chemical potential at integer fillings. This behavior has been attributed to flavor Hund’s coupling arising from the combined effect of on-site inter-flavor Coulomb repulsion and inter-site intra-flavor exchange interactions [29]. Notably, such cascade of phase transitions persists to temperatures above the onset of correlated insulating and superconducting states, indicating the flavor symmetry breaking could be the parent state from which correlated insulating states and superconductivity emerge from.

### Intervalley coherent ground states

Different from a conventional Mott insulator, the flat bands in MATBG are generally described as multicomponent quantum Hall systems with both spin and valley degeneracies, resembling the zeroth Landau level in graphene [30]. Exchange interaction spontaneously drives the system into a subset of the isospin components with complex quantum valley texture in their wavefunction such as the inter-valley coherence (IVC), valley polarization and valley Hall [31,32]. These symmetry-broken quantum valley textures vary among different charge fillings and are susceptible to strain, magnetic field, displacement field, etc.

Encoded with the symmetry-breaking information, intricate quantum valley textures are intimately associated with various correlated phases in MATBG. In particular, the IVC state which means the spontaneous hybridization between inequivalent K and K’ valleys, is predicted to be a candidate ground state for correlated insulators at even integer fillings and the superconducting states [31,33]. With the atomic resolution, STM can be used to distinguish the quantum valley texture by imaging the atomic-scale spatial distribution of electron states. The LDOS measurements at *ν* = ±2 shows real-space features that indicate translation and rotation symmetry-broken Kekulé patterns with a $\sqrt 3 \times \sqrt 3$ super-periodicity on the graphene atomic lattice [25,34], as shown in Fig. 3c. The local magnitude and phase fast Fourier transform (FFT) plots of Kekulé patterns clearly show wavevectors that connect the graphene valleys at K and K’, indicates that the wavefunction at *ν* = ±2 is a coherent superposition in the two valleys, directly signifying the ground state of IVC. The IVC ground state hosts various candidate orders such as the incommensurate Kekulé spiral (IKS), Kramers intervalley coherent (K-IVC) and time-reversal symmetric intervalley coherent (T-IVC) orders. With a symmetry-based approach to extract local order parameters from STM images, order parameters of IVC bond, IVC sublattice A and IVC sublattice B, decomposed from FFT plots of Kekulé patterns (Fig. 3c), exhibit a long-wavelength moiré-scale modulation. This indicates the IKS order that simultaneously breaks both moiré-scale translation and rotation symmetries as the IVC ground state in the typical strained samples (strain *\varepsilon >* 0.1%) [25,34–36]. In ultralow-strain samples (*\varepsilon <* 0.1%) moiré-periodic order parameters show the absence of moiré-scale symmetry breaking, corresponding to the T-IVC ground state.

Remarkably, the Kekulé distortion persists across a range of doping, magnetic fields and temperatures. The coexistence of IVC order with pseudogap behavior and its evolution with carrier density across the superconducting domes suggest that superconductivity may arise from a parent IVC phase, possibly mediated by collective valley-pseudospin fluctuations. Taken together with correlated insulators, these findings provide compelling evidence that intervalley coherent order is a unifying feature of correlated phases in twisted graphene moiré systems, underscoring the central role of the valley degree of freedom in organizing electronic order in graphene flat-band systems.

### Localized moments and topological heavy fermions

Soon after the correlated insulators were found in flat bands of MATBG, the microscopic description of its correlation physics has been much sought after. An exotic finding associated with electronic correlation is the localized moments that arise from isospin symmetry-broken ground states. The first signature of localized moments is the Pomeranchuk effect around integer moiré fillings in MATBG [37,38]. Pomeranchuk effect was manifested as the solidification of liquid ^3^He upon increasing temperature, owing to the large nuclear spin entropy of spatially localized He atoms [39]. As an analogue in MATBG, the Pomeranchuck effect arises from extra entropy of localized and disordered isospin moments at higher temperature which is distinct from the isospin-unpolarized metallic states of itinerant electrons at low-temperature limit. Experimentally, Pomeranchuck effect in MATBG was hinted by the appeared resistive peak of moiré filling *ν* = -1 at high temperature that implies the localization of electrons [37]. Based on electronic compressibility probes like scanning single electron transistor (SET) and capacitance bridges, thermodynamic measurements by relating the entropy *S* to the chemical potential *μ* through Maxwell relations


(3) \begin{eqnarray*}
{{ \left(\frac{{\partial S}}{{\partial \nu }}\right)}_T} = - {{ \left(\frac{{\partial \mu }}{{\partial T}} \right)}_\nu }
\end{eqnarray*}

clearly revealed the extra entropy around 1${{k}_{\mathrm{B}}}$ per moiré unit cell for the localized magnetic moments [37,38]. Additional signatures of localized magnetic moments include the sign reversals of the thermopower near the moiré filling *ν* = ±1, which remain nearly temperature independent from 5 K to 60 K, as well as magnetic-field-induced suppression of the thermopower, resistivity and entropy [40,41].

However, correlated states can exhibit electronic behaviors that are sometimes contradictory to those expected for localized moments. The absence of a thermodynamic gap at most of moiré fillings suggests itinerant electrons are responsible for the strongly correlated phase. In STM measurements, the charge carriers are found to be dispersed around rather than exactly at AA sites of moiré patterns. Most importantly, thermodynamic probes of chemical-potential sensors revealed a cascade of Dirac revival for electrons at each integer filling, pointing to the presence of Dirac-like delocalized electrons [26,27]. On the other hand, the theoretical paradigm of topological Chern band contrasts with the picture of localized moments. Together, these observations indicate that correlated flat bands in MATBG and other related moiré superlattice are governed by a more intricate microscopic mechanism, one that must reconcile the coexistence and competition between itinerant (light) and localized (heavy) electrons.

Theory of topological heavy fermion was proposed to reconcile such disagreement: the flat band described by Bistrtzer-MacDonald model is renormalized by the topology and interactions into a hybridization of localized *f* orbitals and extended topological metallic conduction *c* bands at different momenta [42]. Recently, the quantum twisting microscope (QTM) which probes the momentum-resolved tunneling across the junction between monolayer graphene on the tip and the targeted quantum materials on the rotatable substrate, has been developed to be a high-resolution spectroscopic technique to map the band dispersion in momentum space [43,44]. Cryogenic QTM explicitly revealed the topological heavy fermion-like band structure near magic angle: a gapped flat band across most of momentum space while a gapless and dispersive Dirac-like band at ${\mathrm{\Gamma }}$ point of the mini-Brillouin zone [45] (Fig. 3d). It explained the contradictory characteristics of electronic cascades and Dirac revivals by considering the reshuffling of charge between the localized and delocalized states. To disentangle the hybridized *c* and *f* electrons contributions in transport, photo-thermoelectric experiments, which probe the local Seebeck coefficient, provide a direct measure of electron-hole excitation asymmetry across the correlated gap at integer fillings of the moiré unit cell (Fig. 3e) [46,47]. In the framework of topological heavy fermion, the electron and hole excitations correspond to extended *c* and localized *f* electrons, respectively. The latter has a diminished lifetime and thus less Seebeck effect contribution, accounting for the observed overall negative *S* values in the electron-doped region.

## TOPOLOGY EMERGENT FROM CORRELATED FLAT BANDS

Topology characterized as Berry curvature and Chern number for electronic bands has long been the focus of research on quantum materials. Crystalline graphene has exhibited topological behaviors, such as quantum Hall effect under magnetic field [48,49] or Berry curvature hotspot for valley electrons under broken inversion symmetry [50,51]. Though the Haldane model and Kane-Mele model were first proposed based on monolayer graphene [52,53], the topologically nontrivial Chern band at zero magnetic field has not been realized in graphene system until the advent of MATBG moiré superlattice.

### Orbital Chern insulators

**Figure 4.**
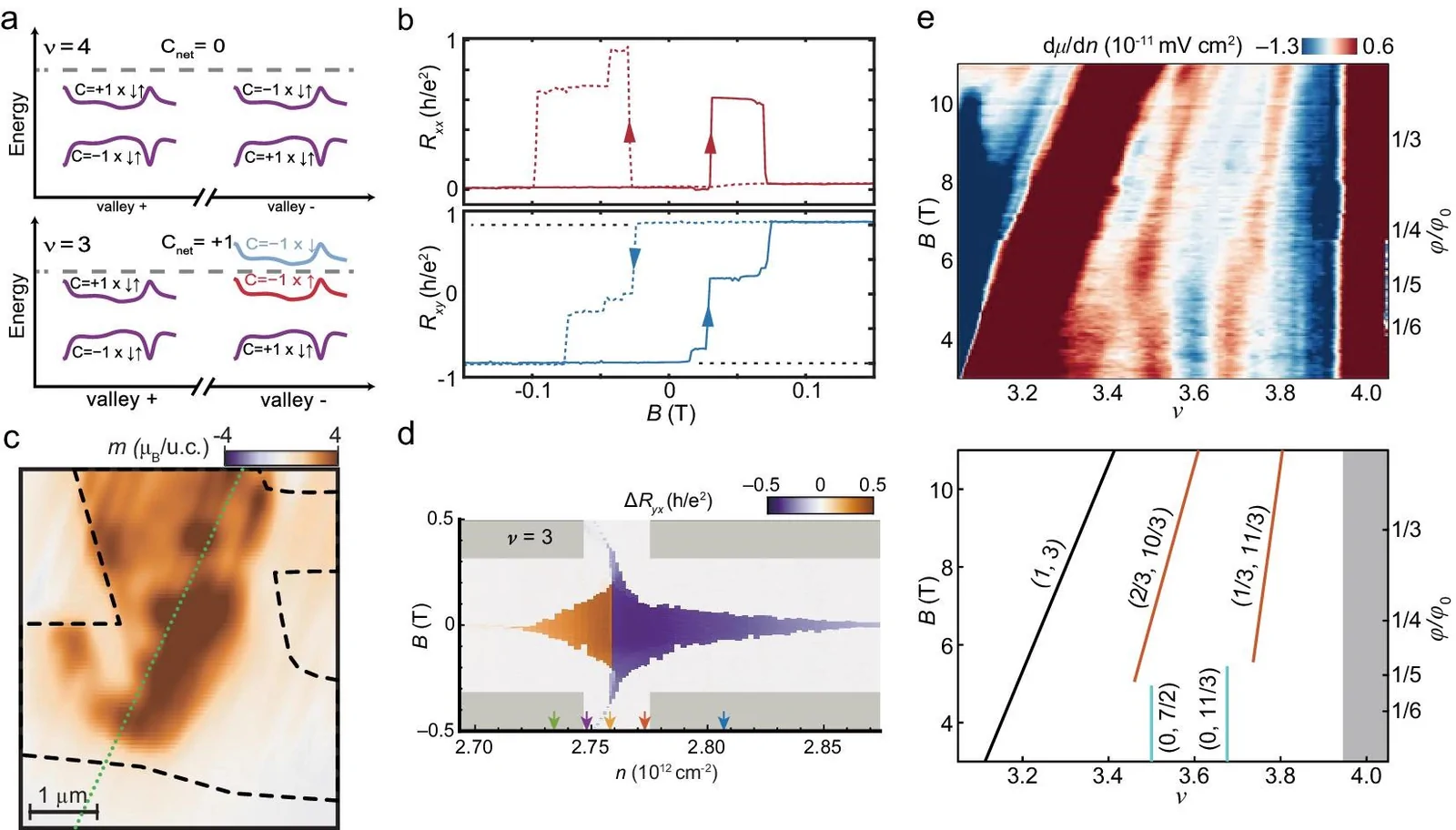
Nontrivial topology of flat band in twisted graphene moiré superlattice: (a) Schematics of band structure at full filling *ν* = 4 and *ν* = 3 [10]. The net Chern number at *ν* = 3 is ${{C}_{\rm net}} = 1$. (b) Quantized anomalous Hall effect characterized by longitudinal resistance ${{R}_{xx}}$ and Hall resistance ${{R}_{xy}}$ at filling factor *ν* = 3 [10]. The arrows indicate sweeping directions of magnetic field. (c) Mapping plot of magnetization density *m*. The black dash lines indicate the edges of the sample [56]. The unit of *m* is Bohr magneton per moiré unit cell (u.c.). (d) Magnetization jump across the Chern insulator gap in twisted monolayer-bilayer graphene [60]. $\Delta {{R}_{yx}} = R_{yx}^{{{B}_ \downarrow }} - R_{yx}^{{{B}_ \uparrow }}$ is the Hall resistance difference between different sweeping directions of magnetic field. (e) Fractional Chern insulators (FCIs) in MATBG [12]. The top panel shows the local inverse compressibility ${\mathrm{d}}\mu /{\mathrm{d}}n$ as a function of magnetic field *B* and filling factor *ν*. In the bottom panel, charge density waves and FCIs are illustrated by the light blue and orange lines, respectively.

By breaking the *C_2z_T* symmetry, the charge neutrality point of MATBG is gapped to form the degenerate conduction and valence valley-projected Chern bands. In case of MATBG alignment with the hexagonal boron nitride substrate, the broken *C_2z_* sublattice symmetry endows the valley-projected flat band with a Chern number *C* = ±1, which is related to two different valleys K and K’ by time-reversal symmetry. The topological Chern flat bands can be regarded as copies of Landau levels at a finite out-of-plane magnetic field. At moiré filling of *ν* = ±1 and *ν* = ±3, exchange interactions drive the system to be a valley-polarized Chern insulators and a net Chern number for the whole system is equal to one (Fig. 4a) [9,10,54]. Consequently, the transverse Hall resistance *R_xy_* is quantized to be *h*/e^2^ and longitudinal resistance *R_xx_* vanishes at zero magnetic field. Both the transverse Hall resistance and longitudinal resistance feature pronounced hysteresis with respect to out-of-plane magnetic field, with the coercive field at the order of 0.1 T (Fig. 4b). This is the so-called quantum anomalous Hall (QAH) effect. The energy gap of the QAH insulator has been found to exceed the Curie temperature, and the Hall resistance remains quantized to within 0.1% of the von Klitzing constant at zero magnetic field, persists to temperatures of several Kelvin [10]. This contrasts with the low temperature and small energy gap of QAH states in magnetic-doped topological insulators (MTIs), where magnetic disorders severely impact the performance of QAH state [55]. However, experimental realization of QAH in hBN-aligned MATBG has so far been limited to a small number of devices [10,54,56], highly depending on the stacking configuration. The absence or fragility of QAH states in hBN-aligned MATBG might originate from the incommensurability between graphene-graphene and graphene-hBN moiré patterns, which generates effective potential disorders that suppress the global percolation of local Chern numbers [57]. Thereby, QAH states are suggested to emerge in a narrow twist-angle window in which two moiré patterns become commensurate.

Different from MTIs where the large spin magnetization of several Bohr magneton ${{\mu }_B}$ per atom is dominate and orbital magnetization is negligible, graphene is not an intrinsically magnetic material and preserves vanishing spin-orbit coupling. As a result, orbital contribution to the magnetic moment can be comparable to the spin in graphene systems. The valley degree of freedom in graphene offers time-reversal-symmetry-related electron species that is separated from the spin. By driving the valley-projected flat bands into a valley-polarized QAH insulator at moiré fillings *ν* = ±1 and *ν* = ±3 with electron interactions, the net nonzero orbital moment is obtained and primarily contributes to the whole magnetization. This is why QAH state in twisted graphene superlattice is also known as “orbital Chern insulators” [56,58]. The orbital ferromagnetism can be assured by measuring the magnetization. For the spin and valley projected flat band, if ferromagnetism is from spin moment, the magnetization density *m* should be 1 ${{\mu }_{\mathrm{B}}}$ per moiré unit cell area. While for orbital moment, the current loop on the moiré length scale generates orbital magnetization of several Bohr magnetons. Magnetometry of nano superconducting quantum interference device (SQUID) scanning local probe has high magnetic field sensitivity (15 nT/Hz^1/2^) and spatial resolution (10-nm scale), providing an accurate measure of magnetization density *m.* Experiments indicate a magnetization density *m* of 2–4 ${{\mu }_{\mathrm{B}}}$ per moiré unit cell (Fig. 4c), in good agreement with the prediction of orbital moment [56]. This result corresponds to magnetization density of 1.8 × 10^−4^ to 3.6 × 10^−4^  ${{\mu }_{\mathrm{B}}}$ per carbon atom, which is distinct from MTIs with a value of several ${{\mu }_{\mathrm{B}}}$ per atom.

In a spin or orbital Chern insulator, the topologically protected edge states also contribute to the magnetization. When the chemical potential *μ* is changed by ${\mathrm{\delta }}\mu$, edge-state contribution of magnetization ${\mathrm{\delta }}M$ is ${\mathrm{\delta }}M/{\mathrm{\delta }}\mu = C{\mathrm{e}}/2\pi \hbar $, where *C* is the Chern number, ${\mathrm{e}}$ is the elementary charge, *ℏ* is the reduced Planck constant. Across the Chern gap ${{E}_{\mathrm{\Delta }}}$, the magnetization jumps by


(4) \begin{eqnarray*}\frac{{\Delta M}}{{{{\mu }_{\mathrm{B}}}/{{A}_{uc}}}} = \frac{{C{{m}_{\mathrm{e}}}{{A}_{uc}}{{E}_{\mathrm{\Delta }}}}}{{\pi {{\hbar }^2}}},
\end{eqnarray*}

where ${{A}_{uc}}$ is the area of unit cell, ${{m}_{\mathrm{e}}}$ is the electron mass [59]. In MTIs, the spin magnetization is around 1 ${{\mu }_{\mathrm{B}}}/{{A}_{uc}}$. As the Chern gap ${{E}_{\mathrm{\Delta }}}$ is on the order of few millielectronvolts, ${{E}_{\mathrm{\Delta }}} \ll {{\hbar }^2}/{{m}_{\mathrm{e}}}{{A}_{uc}}$ since ${{A}_{uc}}$ of MTIs is on the atomic scale. The edge-state contribution of magnetization jumping *Δ M* across the Chern gap is negligible. However, for orbital Chern insulators in moiré superlattice, ${{A}_{uc}}$ is the large moiré unit cell area at the order of 100 nm^2^, therefore ${{E}_{\mathrm{\Delta }}}$  $\approx {{\hbar }^2}/{{m}_{\mathrm{e}}}{{A}_{uc}}$. This results in *Δ M* comparable to the orbital magnetization of several ${{\mu }_{\mathrm{B}}}/{{A}_{uc}}$. Such additional prominent magnetization from edge states in orbital Chern insulators can reverse the sign of magnetization when the chemical potential is swept across the Chern gap with electrostatic gating. Transport and local magnetic imaging probed such electric-field-induced nonvolatile reversal of magnetic states (Fig. 4d) [60], paving the way for application of nonvolatile and ultralow-power magnetic memory devices.

### Fractional Chern insulators

The discovery of Chern insulators in twisted graphene superlattice invoked the immense interest to realize FCIs by fractionalizing the flat Chern bands. FCIs are the lattice analogues of fractional quantum Hall (FQH) states, characterized by spontaneous breaking of both lattice translational and time-reversal symmetries [61–66]. Like FQH states, FCIs could host quasi-particle excitations of anyons that obey fractional statistics. When topological Chern band is gapped at its partially filling *ν* due to electron–electron interactions, FCIs appear if the parent Chern number *C* is also fractionalized into ${{C}_\nu } = \nu C$. Therefore, the formation of FCIs requires a particular quantum geometry of uniform Berry curvature distribution in the parent flat Chern bands. The trials of realizing zero-magnetic-field FCIs in a variety of twisted graphene systems came to failures, as the competed topological-trivial charge density wave states with zero Chern number are mostly favored [12]. Magnetic field is demonstrated to be of critical importance to flatten the Berry curvature distribution in MATBG, leading to the emergence of FCIs in a finite out-of-plane magnetic field (Fig. 4e). Recent progresses in experiments have realized fractional quantum anomalous Hall (FQAH) states—the form of FCIs at zero magnetic field, in other moiré superlattice systems like twisted MoTe_2_ [67–70] and rhombohedral graphene/hBN superlattice [71]. Flat bands of twisted MoTe_2_ and rhombohedral graphene/hBN superlattice have Chern numbers of *C = ± 1*. At zero magnetic field, FQAH states exhibit quantized Hall resistance plateaus of ${{R}_{xy}} = \frac{h}{{\nu {{e}^2}}}$ at fractional fillings of $\nu = \frac{2}{3},\frac{3}{5},$ etc., resembling the Jain sequence of FQH states [72].

### Topological electronic crystals

At fractional filling *ν* of flat bands, Coulomb interaction or an underlying staggered potential can break the continuous or discrete translational symmetry, driving the two-dimensional electron gas (2DEG) into electronic crystal phases such as Wigner crystals or charge density wave (CDW) states. In topologically nontrivial moiré superlattices, if time-reversal symmetry is spontaneously broken, these electronic crystal states may additionally acquire anomalous Hall responses characterized by nonzero Chern numbers [54,73–75].

In contrast to FCIs where the Hall resistance is fractional and directly tied to the filling *ν* (i.e. ${{R}_{xy}} = \frac{h}{{\nu C{{{\mathrm{e}}}^2}}}$), the Chern number ${{C}_\nu }$ of topological electronic crystal (TEC) states is not universally fixed by *ν C*, and can exhibit a variety of behaviors depending on the interplay between interactions and band topology. Recent experiments in graphene moiré systems have revealed several representative cases: (ⅰ) ${{C}_\nu }$ coincides with an integer value consistent with *ν C* when the parent band carries a higher Chern number (*C\not= 1*), such that interaction-driven symmetry breaking gaps out the system without fractionalization (e.g. in twisted monolayer-bilayer graphene [73]); (ⅱ) ${{C}_\nu }$ remains equal to the parent-band Chern number *C* over a finite range of filling, despite the presence of additional carriers (e.g. in MATBG in finite magnetic field [1] and hBN-aligned rhombohedral multilayer graphene [74]); and (ⅲ) ${{C}_\nu }$ deviates substantially from that of the parent band, reflecting a strong reconstruction of the underlying topology (e.g. in twisted bilayer-trilayer graphene [75] and hBN-aligned rhombohedral multilayer graphene [76]).

The second case bears resemblance to the reentrant quantum Hall effect (RQHE) observed at high magnetic fields, where the Hall conductance returns to that of a nearby integer quantum Hall state upon partial filling of higher Landau levels. In conventional RQHE, this behavior is understood as the coexistence of an incompressible integer quantum Hall liquid with a topologically trivial pinned electron solid (such as a Wigner crystal, bubble phase, or stripe phase) formed by the excess carriers [77–79]. By analogy, TEC states in this regime may be interpreted as a coexistence of an integer QAH background and an additional, topologically trivial electronic crystal [74].

However, recent theoretical works suggest a more intrinsic possibility: even in the absence of an external magnetic field, a Wigner crystal formed in a topological band can itself spontaneously break time-reversal symmetry and develop a nonzero integer Chern number, provided that the parent band hosts nontrivial quantum geometry of concentrated Berry curvature [80–84]. This phase, referred to as an anomalous Hall crystal (AHC), can be viewed as a zero-field analog of a Hall crystal [85]. Importantly, the Chern number of an AHC is not simply inherited from the parent band, but instead emerges from the interplay between electron–electron interactions and band topology, corresponding to the third case described above. In general, AHC states are expected to arise at both continuous incommensurate and commensurate fillings of the underlying moiré patterns where electronic interactions—not the moiré potential—drive the spontaneous breaking of translational symmetry. Nevertheless, when the moiré potential plays a significant role, generalized AHC states at discrete commensurate fillings may also be stabilized [75].

## SUPERCONDUCTIVITY: INTERTWINING OF CORRELATION AND TOPOLOGY

**Figure 5.**
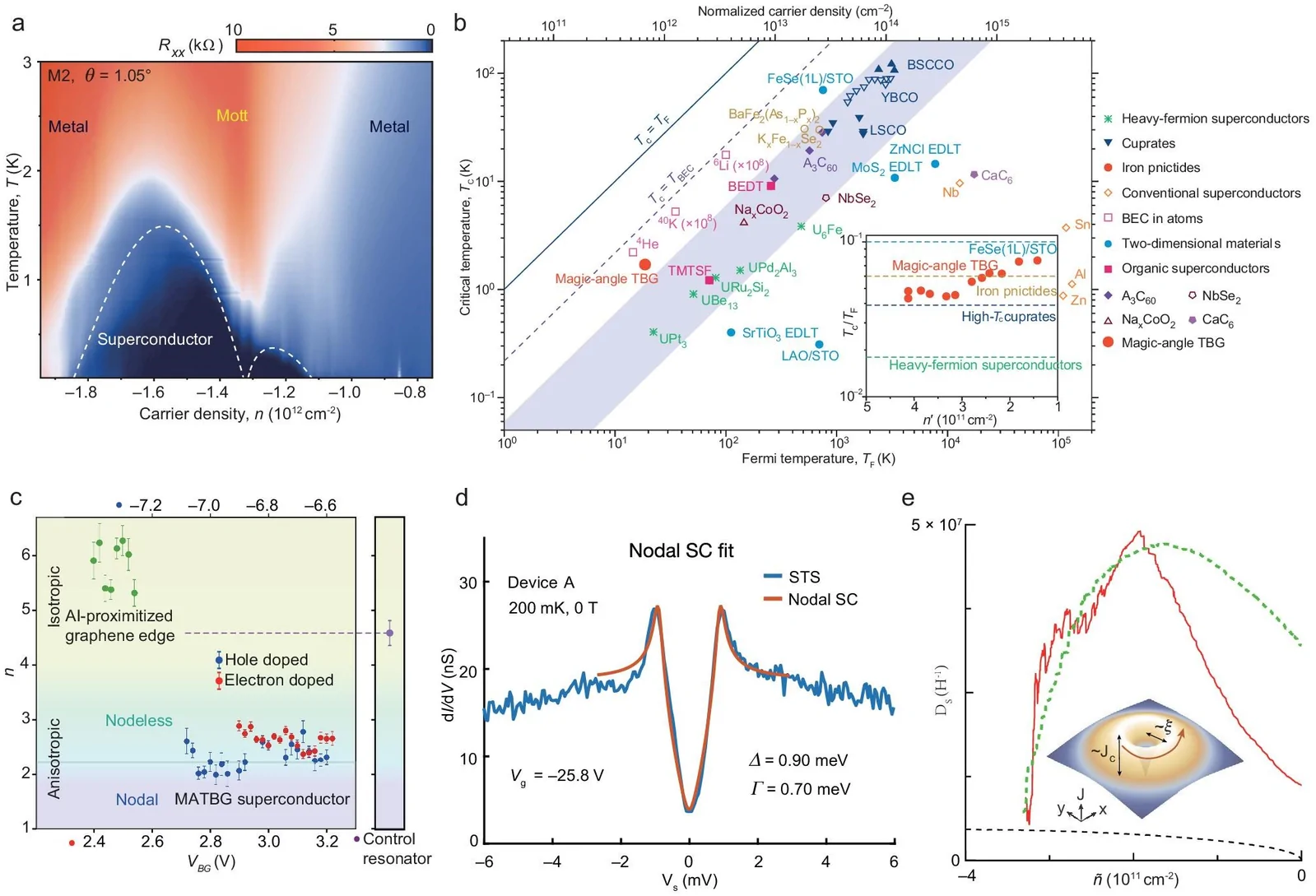
Signatures of unconventional superconductivity in MATBG: (a) Superconducting phase diagram in a carrier density-temperature mapping plot [6]. (b) Logarithmic plot of critical temperature *T*_C_ versus Fermi temperature *T*_F_ for various superconductors, showing the superconductivity in MATBG is in the strong-coupling limit [6]. (c) Power-law fit exponent *n* of the temperature-dependent shift in resonant frequency due to varying superfluid across the entire superconducting dome in both electron and hole-doped regime in MATBG, showing anisotropic superconducting gap in the fermi liquid framework [91]. (d) Tunneling spectrum on MATBG in the superconducting state, showing V-shaped spectra that can be fitted using the model quasiparticle density of states (DOS) for a nodal superconductor [93]. (e) Superfluid weight as a function of superfluid density [87]. The black dash line shows calculated ${{D}_s}$ using the conventional band dispersion relation ${{D}_s}( T ) = {{{\mathrm{e}}}^2}{{n}_s}( T )/{{m}^*}$. The red line and green dashed line denote ${{D}_s}$ extracted with the measured critical supercurrent density and a mean-field theory that considers the interaction-driven quantum geometry.

Superconductivity in twisted bilayer graphene was observed at the magic angle of approximately *θ* ≈ 1.1°[6], with the critical temperature up to 2 K at anomalously low carrier density close to the half filling (Fig. 5a). In this section, we discuss how the correlation and band topology reshape the unconventional superconducting behaviors in MATBG.

### Non-BCS superconductivity

In a conventional weak-coupling BCS superconductor, electrons pair with a coherence length larger than the interparticle distance and at an energy window close to the Fermi level. The phonon-mediated superconducting pairing energy in BCS theory that determines the superconducting transition temperature *T*_C_ is much smaller than the Fermi energy ${{E}_{\mathrm{F}}}$, yielding the result of *T*_C_$\ \ll$  *T*_F_ (here ${{T}_{\mathrm{F}}}$ is the Fermi temperature). Given the extremely low superfluid density (due to the reset state after the half filling) and low Fermi energy (due to the flat band dispersion), superconductivity in MATBG is expected to be strongly coupled. The coherence length (*ξ* ≈ 50 nm at optimal doping) and averaged interparticle distance are found to be at the same order, revealing the superconducting state in MATBG appears in the strong-coupling regime of the BCS to Bose–Einstein condensate (BEC) crossover. In the Uemura plot which compares the superconducting transition temperature (*T*_C_) to the estimated Fermi temperature (*T*_F_), conventional weak-coupling BCS superconductors exhibit ratio of *T*_C_/*T*_F_  *≪* 1, as shown in Fig. 5b whereas most unconventional superconductors, such as cuprates, heavy fermion systems, and organic materials fall in the range of 0.01–0.05 [86]. MATBG shows a *T*_C_/*T*_F_ ratio of about 0.08 at optimal doping, suggesting that its superconductivity likely arises from electron correlation rather than merely from conventional phonon-mediated BCS pairing [6,87].

Other unconventional features further distinguish the superconducting state of MATBG from that of conventional isotropic BCS superconductors. One prominent example is the emergence of electronic nematicity in both the normal and superconducting state, arising from the spontaneous breaking of the underlying lattice symmetry due to electronic correlation [88]. In alternating magic angle twisted trilayer graphene (MATTG), the preferred direction of superconducting transport aligns with the principal axis of the metallic phase that has the highest resistivity, while the strange metal behavior is oriented along the principal axis with the lowest resistivity [89]. In addition, MATTG also exhibits superconductivity that violates the Pauli limit by a factor of 2–3, indicating possible spin-triplet superconductivity [90].

Further evidence for possible unconventional superconductivity in MATBG and MATTG was obtained through microwave circuit quantum electrodynamics, where superfluid stiffness ${{\rho }_s}$ can be measured via the probed kinetic inductance ${{L}_K}$ [91,92]. In superconductors, the superfluid stiffness ${{\rho }_s}$ is intimately connected to the superconducting pairing symmetry and gap structure due to quasiparticle spectrum at a finite temperature. The observed power-law temperature dependence of ${{\rho }_s}$ and nonlinear Meissner effect in the current-bias dependence contradicts with the exponential dependence in isotropic *s*-wave superconductors, as shown in Fig. 5c, indicating an anisotropic superconducting gap in MATBG [91]. Both signatures indicate nodal structures in the superconducting order parameter, in contrast to conventional BCS superconductors where the strength of Cooper pairing is characterized by the isotropic superconducting gap in momentum space.

Other than conventional electrical transport measurements, STM measurements in MATBG and MATTG systems have also provided compelling evidence for anisotropic nodal superconductivity, pointing toward an unconventional superconducting pairing mechanism [93,94]. In MATBG, tunneling spectra near half-filling reveal a distinct V-shaped gap structure in the differential conductance, shown in Fig. 5d, indicative of nodes in the superconducting gap function, where quasiparticle excitation remains gapless along specific momentum directions. In MATTG, STM uncovers an intriguing evolution from a U-shaped to a V-shaped gap as the filling is tuned away from *ν* ≈ −2 toward *ν* ≈ −2.3. This transformation signals a change from a fully gapped state to gapless paired states, or a transition from BEC and BCS phases with a single nodal order parameter [94]. Moreover, both systems exhibit a pseudogap regime above the superconducting transition temperature as well as particle-hole asymmetry, further support the role of strong electronic interactions and spontaneous symmetry breaking. Recently, combined tunneling spectroscopy and transport measurements on MATTG reveal a V-shaped tunneling gap that directly links to the superconducting state observed in transport. The tunneling spectra show a linear gap-filling with increasing temperature and magnetic field, consistent with a nodal superconducting order parameter [95]. Crucially, these phenomena are highly sensitive to the relative alignment between the graphene layers and hBN, consistent with observations in transport measurements [9,10]. Both the pseudogap and superconductivity are absent when MATBG is commensurately aligned with the hBN substrate, suggesting that the structural characteristics and/or the *C_2z_T* symmetry of unaligned MATBG are required for stabilizing these ground states. On the other hand, misalignment appears to preserve or even enhance nodal superconducting features, likely by preserving underlying symmetries or enhancing valley coherence. These findings point to a rich interplay between twist angle, band topology, substrate alignment, and electron interactions in shaping the symmetry and superconducting order parameter in moiré graphene systems.

### Quantum-geometry enabled superconductivity

The superfluid weight ${{D}_s}$, which characterizes the superfluid phase stiffness in a superconductor, is conventionally believed to be associated with electronic kinetic energy, i.e. the band dispersion. In a parabolic band dispersion, the superfluid weight contributed by electronic kinetic energy is approximated to be:


(5) \begin{eqnarray*}
{{D}_s} = {{D}_{s,\mathrm{disp}}}( T) = {{{\mathrm{e}}}^2}{{n}_s}( T)/{{m}^*},
\end{eqnarray*}

where ${{n}_s}( T )$ is the superfluid density and ${{m}^*}$ is the effective mass of carriers. Since Fermi velocity is vanishing and ${{m}^*}$ is extremely large in flat bands, the superfluid weight ${{D}_s}$ of MATBG is expected to be small. Small ${{D}_s}$ yields a low upper bound of Berezinskii–Kosterlitz–Thouless (BKT) transition temperature ${{T}_{{\mathrm{BKT}}}}$ according to the Nelson–Kosterlitz criterion:


(6) \begin{eqnarray*}\frac{{{{\hbar }^2}{{D}_s}\left( {T = 0} \right)}}{{8{{{\mathrm{e}}}^2}{{k}_{\mathrm{B}}}}} \ge \frac{{{{T}_{{\mathrm{BKT}}}}}}{\pi },
\end{eqnarray*}

where ${{k}_{\mathrm{B}}}$ is the Boltzmann constant [96]. Through Schwinger-limited nonlinear transport analysis from which the effective mass ${{m}^*}$ and thus ${{D}_{s,\mathrm{disp}}}$ can be obtained, the estimated upper bound of ${{T}_{{\mathrm{BKT}}}}$ is much smaller than experimental results [87]. The association between superconductivity and ${{D}_s}$ can be also reflected through the behavior of critical 2D supercurrent density ${{J}_{\mathrm{c}}}$ and kinetic inductance ${{L}_{\mathrm{K}}}$ according to the following relations:


(7) \begin{eqnarray*}
{{D}_s}\left( {T = 0} \right) = \frac{{2\pi {{J}_{\mathrm{c}}}\xi }}{{{{{\mathrm{\Phi }}}_0}}},
\end{eqnarray*}

(8) \begin{eqnarray*}
{{L}_{\mathrm{K}}} = 1/{{D}_s},
\end{eqnarray*}

where ${{{\mathrm{\Phi }}}_0} = h/2{\mathrm{e}}$ is the flux quantum. ${{D}_s}$ is experimentally obtained in superconducting twisted graphene systems via measurements of critical supercurrent density ${{J}_{\mathrm{c}}}$ and kinetic inductance ${{L}_{\mathrm{K}}}$ [87,91,92,97]. However, both probes show much larger values of ${{D}_s}$ than those derived only from the flat band dispersion (Fig. 5e).

This discrepancy is now understood in terms of the superfluid weight ${{D}_s}$, which depends on both the band dispersion and the quantum geometry of the electronic states [98–100]. The total superfluid weight ${{D}_s}$ is expressed as ${{D}_s} = \ {{D}_{s,\mathrm{disp}}} + {{D}_{s,\mathrm{geom}\ }}$where ${{D}_{s,\mathrm{geom}\ }}$arises from nontrivial quantum geometry and is proportional to the quantum metric in a multiband system. This quantum metric manifests as the overlapping of Wannier functions in neighboring lattices and hence enables the transport of quasiparticles like the Cooper pairs. In MATBG, flat bands exhibit highly nontrivial band topology with nonzero Chern number and quantum metric, creating extra ${{D}_{s,\mathrm{geom}\ }}$ contribution and pronounced ${{D}_s}$ even when its conventional dispersion part ${{D}_{s,\mathrm{disp}}}$ is strongly suppressed. Superconductivity in MATBG is therefore proposed to be enhanced by nontrivial quantum geometry of flat bands, even though their flatness would otherwise favor Cooper-pair localization. The contribution of quantum geometry to *T*_C_ can account for the large *T*_C_/*T*_F_ ratio that violates the noninteracting BCS theory.

## MOIRÉ FLAT BANDS BEYOND MATBG

The emergence of correlated states in MATBG has spurred extensive exploration of flat bands graphene-based twisted systems. In particular, the twisted multilayer graphene (TMG) superlattices, formed by integrating additional twisted layer, or altering the “parent” Dirac cones, can serve as a new playground of electronic correlation, superconductivity and band topology. A fundamental distinction from MATBG is that TMG hosts more tunable and markedly different moiré flat bands, arising from the modified symmetry constraints and more complex interlayer couplings associated with additional layers. Here, we summarize several widely studied TMGs systems, briefly outlining their low-energy band structures and highlighting key features of their electronic correlation, superconductivity, and topology.

### Twisted M + N multilayer graphene

**Figure 6.**
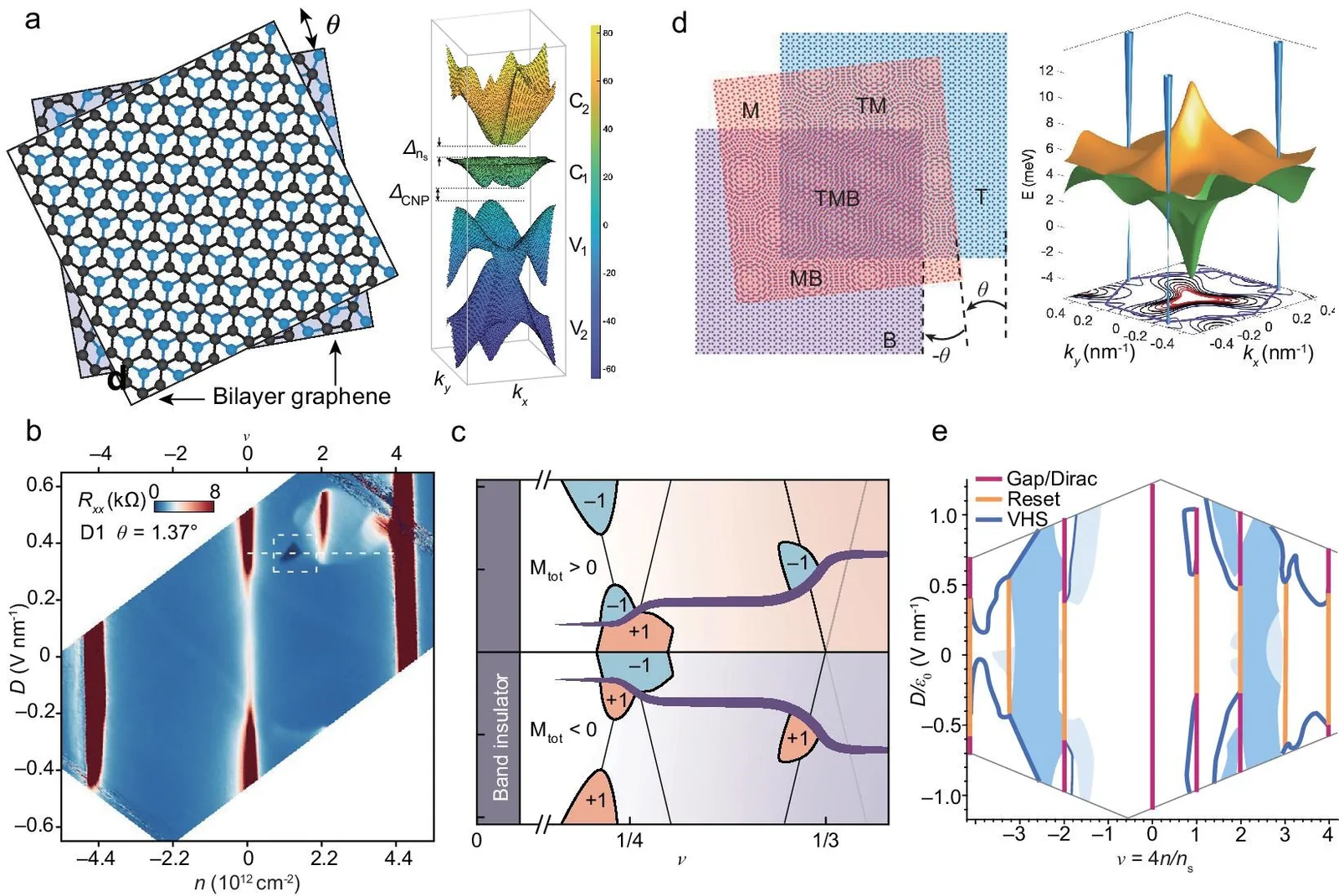
Band structure and electronic properties of twisted multilayer graphene: (a) Schematic of TDBG and calculated band structure at an optimal displacement field and twist angle 1.33° [103]. The C_1_, C_2_ (V_1_, V_2_) denote the first and second conduction (valence) band, respectively. The first conduction band C_1_ is flat and isolated from other bands. (b) Resistivity of 1.37° TDBG proximitized to WSe_2_ as a function of top and bottom gates [108]. The half filling (*ν* = 2) of C_1_ band shows resistive state at optimal displacement field where both charge neutrality point (CNP, *ν* = 0) and full filling of C_1_ band (*ν* = 4) are gapped. Superconductivity appears in small pockets of the phase diagram near the van Hove singularities. (c) Schematic phase diagram of topological Wigner crystal in twisted bilayer-trilayer graphene [75]. The vertical axis denotes perpendicular magnetic field. The Chern numbers at fractional filling factors *ν* = 1/4 and *ν* = 1/3 are *C* = ±1 and tuned by perpendicular magnetic field. (d) Schematic of ATTG and calculated band structure at zero displacement field [111]. (e) Schematic phase diagram of ATTG [110]. The dark blue shadings denote superconducting phase which is bounded by the van Hove singularity denoted by the blue lines.

Inherited from the electrically tunable band structure of the parent crystalline graphene, twisted double layer system of crystalline M-layer and N-layer graphene can exhibit flat bands with a tuning knob of displacement field *D*, as induced by the broken inversion symmetry [101,102]. The widely investigated twisted M + N graphene includes twisted double bilayer graphene (TDBG, 2 + 2), twisted monolayer-bilayer graphene (TMBG, 1 + 2), etc. [103–106]. The displacement field can effectively change the bandwidth and adjust the energy of van Hove singularity where the DOS is divergent, producing a rich phase diagram in *n-D* space (here *n* is the carrier density). In a specific range of displacement field *D*, the low-energy conduction band is flat and well isolated from other bands by gap openings at charge neutrality and full fillings (Fig. 6a). Electronic correlated insulators appear consequently (Fig. 6b). The half-filling correlated insulator in TDBG or TMBG can be different from MATBG, showing a spin-polarized behavior under in-plane magnetic field [103–105]. With an optimal doping away from the half filling, the resistance of TDBG exhibits an abrupt drop as the temperature is lowered, which is believed to be associated with spontaneous symmetry breaking rather than superconductivity [107]. TDBG in proximity to a sheet of tungsten diselenide (WSe_2_) exhibits superconductivity near the van Hove singularities of conduction and valence bands (Fig. 6b) [108].

A large family of twisted M + N multilayer graphene superlattices has been found to exhibit exotic anomalous Hall (AH) or QAH effects that are markedly different from MATBG [102]. In twisted bilayer-trilayer graphene superlattice, TEC states at zero magnetic field are demonstrated in a general formation where discrete rather than continuous translation symmetry is broken (Fig. 6c) [75]. In addition, since twisted M + N multilayer graphene has the intrinsic *C_2z_* sublattice symmetry breaking, the AH or QAH effect appears without additional alignment of graphene layers to the hBN substrate [60,73,106]. Another completely different feature from MATBG is the higher Chern numbers in twisted M + N graphene, which is of high importance to establish FQAH states that do not resemble the FQH state—a typical counterpart of FQAH in high magnetic field. TMBG exhibited Chern number *C* = ±2 for spin and valley projected flat bands. At moiré fillings of *ν* = 1 and *ν* = 3, QAHE with Hall resistance approximately equal to *h*/2*e*^2^ were observed. Fractionalizing the spontaneous valley-polarized flat bands at fractional fillings of *ν* = 3/2 results in the topological charge density wave state with Chern number *C* = 1 in TMBG—an electronic state with both the chiral edge modes and unit cell doubling [73].

### Alternating twisted multilayer graphene

One family of graphene moiré system that mostly resembles MATBG is the alternating twisted multilayer graphene (ATMG), where monolayer graphene layers (layer number *N* > 3) are successively stacked with alternating twist angle *θ* and *- θ*. As the even or odd-numbered layers are strictly aligned, ATMG hosts *C_2z_* symmetry. Unlike most of other moiré systems, ATMG has [*N*/2] bands at the lowest energy which can be flattened at the corresponding magic angle


(9) \begin{eqnarray*}
\theta _{{\mathrm{magic}}}^{{\mathrm{ATMG}}} = 2\theta _{{\mathrm{magic}}}^{{\mathrm{TBG}}}\cos \left( {\frac{{\pi k}}{{N + 1}}} \right),
\end{eqnarray*}

where *k* = 1, 2, 3…[*N*/2], [*N*/2] is the largest integer less than *N*/2, and $\theta _{{\mathrm{magic}}}^{{\mathrm{TBG}}} \approx 1.1$° is the magic angle of TBG [109]. When *N* is odd, there is a dispersive Dirac cone concomitant with the flat bands. For instance, in alternating twisted trilayer graphene, the low-energy band structure is decomposed into a MATBG-like nondispersive band with the scaled interlayer coupling by a factor of *\sqrt 2*, and dispersive Dirac cones with Dirac point located at K points in the moiré Brillouin zone [110–112] as shown in Fig. 6d. The TBG-like band is extremely flat at $\theta \approx 1.5$° that can host pronounced electronic correlation effects like in MATBG [112,113]. ATTG has a mirror symmetry when the displacement field *D* is zero. With an applied external displacement field to break the mirror symmetry, the electronic potential difference shifts Dirac cones up in energy and hybridizes them into the flat band sector [112].

Of the critical importance, ATMG can have larger magic twist angles which can mitigate one of the most severe experimental challenges—twist angle control. It has been revealed to be more structurally stable than MATBG, enabling more robust superconductivity and higher *T*_C_ [110,111,114,115]. Similar to MATBG, alternating twisted trilayer graphene also exhibits multiple features of unconventional superconductivity, as discussed in Section 5. In its *n-D* phase diagram, the superconductivity is connected to the symmetry-broken phase and bounded by the van Hove singularity, which cannot reconcile with the weak-coupling BCS theory (Fig. 6e) [110].

### Moiré quasicrystal and supermoiré superlattice

Beyond the single periodical moiré pattern, the moiré design now has expanded to the multi-moiré patterns by constructing multiple twist angles that play with the stacking chirality and angular difference. The representative case is twisted trilayer graphene with two twist angles ${{\theta }_{{\mathrm{TM}}}}$ and ${{\theta }_{{\mathrm{MB}}}}$ (here, ${{\theta }_{{\mathrm{TM}}}}$ and ${{\theta }_{{\mathrm{MB}}}}$ are twist angle between the top and middle layers, middle and bottom layers, respectively). When the ratio ${{\theta }_{{\mathrm{TM}}}}/{{\theta }_{{\mathrm{MB}}}}$ is away from plus or minus one, two mutually incommensurate moiré patterns will lead to a quasiperiodic structure, named moiré quasicrystal. Moiré quasiperiodicity is defined on the moiré length and doesn’t exhibit rotation symmetries like in the usual quasicrystal. Recent experiments and theoretical predictions point to the presence of flat bands that can induce electronic correlation and superconductivity in twisted trilayer graphene moiré quasicrystals [116]. When ${{\theta }_{{\mathrm{TM}}}}$ and ${{\theta }_{{\mathrm{MB}}}}$ have the same sign, namely graphene is helically stacked, or ${{\theta }_{{\mathrm{TM}}}} \approx - {{\theta }_{{\mathrm{MB}}}}$ and ${{\theta }_{{\mathrm{TB}}}} \ll - {{\theta }_{{\mathrm{TM}}}}$, numerical calculations and experiments indicate the presence of long-wavelength supermoiré structure arising from the interference of the two moiré patterns. The supermoiré effect can impact strongly on the electronic correlation and band topology, leading to symmetry-broken ground states and anomalous Hall effect [117,118].

## OUTLOOK

Despite significant progress in revealing the exotic flat-band physics in twisted graphene superlattice, several key challenges remain. A primary obstacle is that the electronic states in twisted graphene are susceptible to twist angle inhomogeneity and strain, leading to difficulties in reproducing these states in different devices. A reliable control of the twist angle and the suppression of twist-angle disorder is therefore of high importance for the ongoing experimental studies of twisted graphene.

The microscopic superconducting pairing mechanism of twisted graphene remains enigmatic. Recent studies showed that when a metallic gate, bilayer graphene or SrTiO₃ dielectric substrates with a large *in-situ* tunable dielectric constant are placed close to TBG to enhance the screening effect and reduce Coulomb interactions, the correlated insulating phases are suppressed while superconductivity remains robust, suggesting that superconducting states are independent of and probably competing with the nearby correlated insulators [119–122]. Such behaviors are distinct from high temperature cuprate superconductors where the antiferromagnetic Mott insulator is a “parent” state of superconductivity. Indeed, more and more results suggest that superconducting states and correlated insulators in twisted graphene may slightly differ such as in the specific IVC quantum textures [25]. One interpretation for the decoupled superconductivity and correlated insulator is that electron–phonon coupling, amplified by the enhanced density of states in flat bands, plays a central role in the superconducting pairing mechanism [123,124]. While in TBG/SrTiO₃ devices, superconducting pairing mechanism is found to arise from Coulomb interactions given the complete suppression of superconductivity with increasing dielectric screening strength [123]. These experimental results, together with the discussed non-BCS pairing behaviors hint that the microscopic superconducting pairing mechanism in twisted graphene is complex, and both electron-phonon coupling and electron–electron interactions are important for superconducting pairing. Examining the response of superconducting critical temperature *T*_C_ to carbon isotope effect where substitution of ^12^C with heavier ^13^C shifts the phonon spectrum without altering the electronic structure, would provide direct evidence for the role of electron–phonon coupling in the superconducting pairing mechanism.

On the other hand, experimental signatures of anisotropic superconducting gap pose an urgent requirement for a thorough investigation into the symmetry of superconducting order parameter ${\mathrm{\Psi }} = |\Delta ( k )|{{e}^{i\phi ( k )}}$, which is typically subject to the lattice symmetry, spin structure and Coulomb interactions. This requires momentum-resolved and phase-sensitive probes to detect the momentum dependence of superconducting gap *Δ ( k )* and phase change *ϕ ( k )* at a sub-millielectronvolt energy and micrometer spatial scale, imposing great challenges to the traditional spectroscopy techniques. Cryogenic QTM in this context, and phase-sensitive global transport measurements including Little-Parks oscillations in mesoscopic rings, phase-sensitive SQUID interferometry, etc., are hoped to provide more insight.

The future of twisted graphene superlattice also lies in further leveraging designable freedoms with tailored correlations and topology. By integrating tunable strain strength, researchers can control the competing orders and band topology that are linked to quantum textures of many-body wavefunctions [32]. Twisted graphene superlattice holds great promise for exploring quantum phase transitions and exotic quantum criticality beyond conventional paradigms with its flexible *in-situ* tuning knobs. In addition, incorporating exotic graphene stacking order, moiré periodicity and layer symmetry to expand the family of twisted graphene, opens exciting prospects to investigate the unexplored quantum states. The recently investigated twisted rhombohedral graphene provides such opportunities to explore high integer Chern number or novel fractional Chern insulating states outside the traditional Landau-level framework [125–128]. We believe continuous exploration of twisted graphene superlattices promises to uncover surprising physical phenomena and provide new routes toward correlated and topological quantum matter.
